# Recent developments in the diagnosis and management of tuberculosis

**DOI:** 10.1038/npjpcrm.2016.78

**Published:** 2016-11-03

**Authors:** Giorgia Sulis, Rosella Centis, Giovanni Sotgiu, Lia D’Ambrosio, Emanuele Pontali, Antonio Spanevello, Alberto Matteelli, Alimuddin Zumla, Giovanni Battista Migliori

**Affiliations:** 1 Department of Infectious and Tropical Diseases, WHO Collaborating Centre for TB/HIV and TB elimination, University of Brescia, Brescia, Italy; 2 WHO Collaborating Centre for TB and Lung Diseases, Maugeri Institute, IRCCS, Italy; 3 Clinical Epidemiology and Medical Statistics Unit, Department of Biomedical Sciences, University of Sassari—Research, Medical Education and Professional Development Unit, AOU Sassari, Sassari, Italy; 4 Public Health Consulting Group, Lugano, Switzerland; 5 Department of Infectious Diseases, Galliera Hospital, Genova, Italy; 6 Pneumology Unit, Maugeri Institute, IRCCS, Tradate, Italy; 7 Department of Clinical and Experimental Medicine, University of Insubria, Varese, Italy; 8 Division of Infection and Immunity, University College London and NIHR Biomedical Research Centre, UCL Hospitals NHS Foundation Trust, London, UK

## Abstract

Tuberculosis (TB) is a major public health issue worldwide, with ~9.6 million new incident cases and 1.5 million deaths in 2014. The End-TB Strategy launched by the World Health Organization in the context of the post-2015 agenda aims to markedly abate the scourge of TB towards global elimination, by improving current diagnostic and therapeutic practices, promoting preventative interventions, stimulating government commitment and increased financing, and intensifying research and innovation. The emergence and spread of multidrug-resistant strains is currently among the greatest concerns, which may hinder the achievement of future goals. It is crucial that primary healthcare providers are sufficiently familiar with the basic principles of TB diagnosis and care, to ensure early case detection and prompt referral to specialised centres for treatment initiation and follow-up. Given their special relationship with patients, they are in the best position to promote educational interventions and identify at-risk individuals as well as to improve adherence to treatment.

## Introduction

Tuberculosis (TB) is still a first-class public health priority, representing the leading cause of death at global level. According to the World Health Organization (WHO), as many as 9.6 million new incident cases and 1.5 million deaths are estimated to have occurred in 2014, with Africa and Asia carrying the greatest burden. Of note, nearly half of the global TB cases are reported in only three countries (India, Indonesia and China).^1^

TB is a well-known poverty-related disease, often associated with social marginalisation and financial deprivation.^2,3^ However, the most important risk factor for TB remains the human immunodeficiency virus (HIV), with a significant proportion of HIV/AIDS mortality still attributable to TB.^4–6^ About 12% of all TB patients were estimated to be HIV infected in 2014, and 25% of TB deaths occurring in the same year involved people living with HIV.^1^ Sub-Saharan Africa accounts for the largest proportion of TB/HIV cases worldwide, in spite of the increasing antiretroviral treatment coverage as well as improvements in access to care in the most affected countries.^7–11^

Non-communicable diseases such as diabetes mellitus are gaining attention as potential risk factors for developing active TB in both poor and wealthy settings, thus raising new issues on prevention and early diagnosis.^12,13^

In spite of its considerable global impact, TB is preventable and curable that makes the fight against the disease a highly cost-effective intervention.^14^ However, the emergence and spread of multidrug resistances may hinder the achievement of goals, as the detection and treatment of multi- and extensively drug-resistant TB (M/XDR-TB) are still a major concern.^15,16^

Around TB, control and elimination programmes have been created, requiring a coordinated effort of both clinicians and public health officers, to ensure that infectious cases are diagnosed and treated rapidly, and infected individuals belonging to high-risk groups are adequately managed.

The aim of this review paper is to provide an overview and elimination strategies, and to describe the basics of present and future challenges in TB diagnosis, treatment and prevention under a clinical perspective.

## TB control from the beginning to the End-TB Strategy

The global agenda beyond 2015 stands on seventeen Sustainable Development Goals (SDGs), one of which (SDG 3) is specifically addressed to health issues and calls for ending the major global epidemics including that of TB.^17,18^ The WHO’s End-TB Strategy, that was approved by the World Health Assembly in May 2014 and further developed throughout 2015 to come into effect in January 2016, clearly points to the global elimination of TB.^19^

However, it took over 20 years to get to this point at strategic level, after achieving several successes in the fight against the disease in the most affected areas.^14^ The recognition of TB as a global emergency only came in 1993 when the WHO first declared the need for great efforts and strong commitment at all levels to address the epidemic.^20,21^ About a year later, the first TB-control strategy (called directly observed therapies) was launched. The adoption and adaptation of this framework in nearly all countries led to a significant improvement in case detection and to the scale-up of a standardised anti-TB treatment.^22^ The constitution of the Global Fund in 2002 contributed to further promote the implementation of this strategy at national level by guiding an appropriate allocation of available funds towards the expansion of access to diagnosis and care. Of note, the Global Fund currently accounts for as much as 72% of the total financing for TB-related interventions, followed by the government of the United States (14%), other governments (8%) and other multilateral organisations (6%).

In 2006, the Stop-TB Strategy replaced the former one by strengthening its basic components to reach more ambitious targets within the following decade.^14,23,24^ Special attention was reserved to TB/HIV co-infection that required a concerted action together with the HIV/AIDS programme, as well as to the rapidly emerging MDR-TB epidemic. The main objectives were to reverse the trend in TB incidence and to halve TB prevalence and mortality worldwide by 2015 as compared with that in 1990. According to the current estimates, all these goals have been fully met, although the declining rate of TB incidence is still too slow (~1.5–2% per year on average).

It is in this scenario that the End-TB Strategy was finally designed, thus bringing a considerable change in the approach to disease control, by establishing increasing links with the socioeconomic and political dimensions of the problem.^19^ The new goals are far more ambitious than the previous ones and point to the global elimination of the disease, starting from low-income countries that are closer to the target. This vision stands on the growing awareness that TB cannot be eliminated from a single area unless all the countries collaborate together towards a shared objective and through a common strategic plan. This approach is very well summarised in the three basic pillars of End-TB: (1) promote integrated patient-centred care and prevention; (2) foster bold policies and supportive systems; and (3) encourage intensified research and innovation. The goals for 2035 include a 95% reduction in TB-related mortality and 90% decline in TB incidence (corresponding to <10 cases per 100,000 population globally), alongside with the complete abolition of catastrophic costs for TB-affected patients and their families.^19^

For the first time in the history of TB-control strategies, a great emphasis is put on the importance of research, which is definitely the key to changing the course of the epidemic; without new diagnostics, new drugs and effective vaccines, the global fight against TB is bound to fail.^25^

Another critical novelty of this post-2015 agenda lies in the pre-eminent role of prevention, although prompt diagnosis and treatment remain the major priority, especially in high TB-burden settings. In accordance with this concept, a specific framework for TB elimination from low-incidence countries was developed besides the End-TB strategy, aimed at stimulating governments and national health authorities to take action against the submerged reservoir of latent TB infection (LTBI).^26–28^

A recent study conducted in Europe has demonstrated that management of LTBI is still far to be applied as per national and international recommendations.^28^ General practitioners have a pre-eminent role in promoting screening for LTBI among contacts of TB cases and other at-risk populations, and treatment of latently infected individuals within the patient-centred vision recently promoted by the International Standards for Tuberculosis Care (ISTC) and European union Standards for Tuberculosis Care (ESTC).^29–31^

It is important to underline that the End-TB Strategy is aimed at serving all countries, although the WHO LTBI guidelines target high- and middle-income countries, and the TB Elimination framework is addressed to low-incidence countries. In this context, we observe low-income countries facing the elimination phase (e.g., Cuba) and European Union countries (e.g., Romania) having medium-level incidence.

## TB diagnostics: past, present and future

TB should be suspected when at least one of four suggestive symptoms is reported (long-lasting fever, cough of 2-week duration or more, night sweats and weight loss). Epidemiological aspects, such as history of contact with a pulmonary TB case and/or exposure to other risk factors for TB acquisition or reactivation, also need to be carefully evaluated.^32^ Special attention should be devoted to patients affected by chronic conditions such as diabetes mellitus who may experience milder symptoms that can be often misinterpreted as related to their underlying disease. The diagnosis of TB must be confirmed by detecting the causative agent (*Mycobacterium tuberculosis*, MTB) in an appropriate biological specimen.

**Figure 1:**
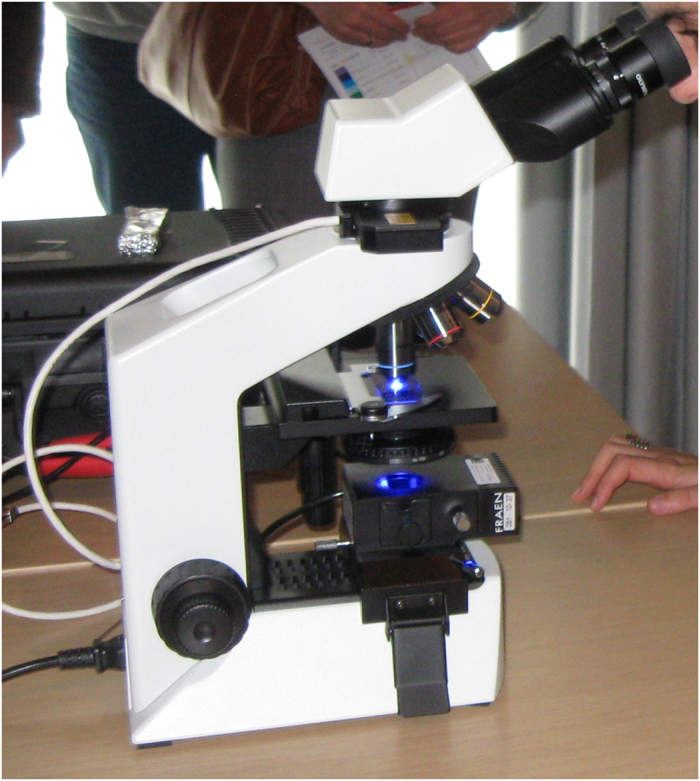
Light-emitting diode fluorescence microscopy.

**Figure 2:**
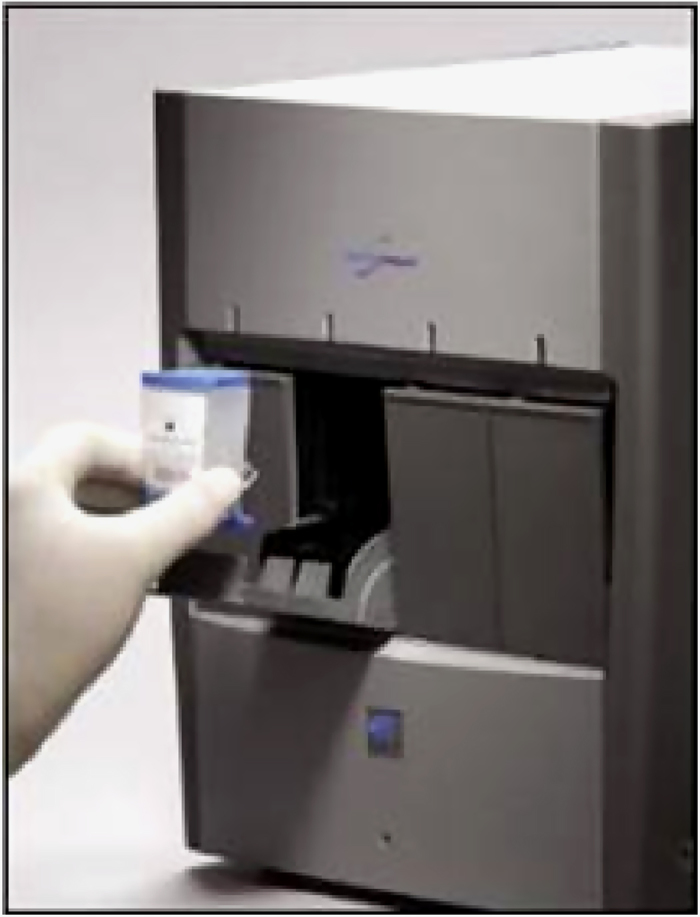
Xpert MTB/Rif.

Direct microscopic examination is a fast and inexpensive method to identify acid-fast bacilli through the use of Ziehl–Neelsen staining; however, it is limited by poor sensitivity and the inability to discriminate between mycobacterial species, which can be a relevant issue especially among children and immunocompromised individuals. Fluorescence or light-emitting diode microscopy may be an alternative to traditional microscopy with a moderate improvement in sensitivity (+10%) but also slightly higher costs and the need for well-trained technicians (Figure 1).^33^ The poorest performances of microscopic examination for the diagnosis of TB are usually observed in high-risk groups such as people living with HIV who often yield a false-negative result. For this reason, WHO currently recommends a biomolecular test as the initial diagnostic tool in case of TB suspect.^34,35^ Among the several commercially available nucleic acid amplification tests, Xpert MTB/Rif (Cepheid, Sunnyvale, CA, USA) is the most efficient and suitable for implementation in resource-constrained settings (at the point-of-care level), as it does not require any sophisticated laboratory facility, is fully automated and provides the results in <2 h (Figure 2).^36^ As compared with smear microscopy, it is characterised by higher sensitivity and specificity on both respiratory and extrapulmonary specimens, and it allows to identify MTB and detect mutations associated to rifampicin resistance, which makes it a good proxy for the detection of MDR strains.^37–39^

Nevertheless, culture remains the gold standard technique for the diagnosis of TB, even though its large-scale use is limited by the long waiting time due to the slow growth rate of mycobacteria (~2–6 weeks in liquid media) with consequent diagnostic delays; furthermore, the need for biosafety level 3 laboratory infrastructures and highly skilled laboratory technicians who are often lacking especially in resource-constrained settings is another significant limitation.^40^ Drug susceptibility testing on solid or liquid culture is essential to confirm a diagnosis of MDR-TB, but its availability in low-income and high-burden areas is far less than is needed. Molecular tests, such as GenoType MTBDRplus, a line probe assay, that rapidly detects TB bacteria and identifies resistances to the most important first-line drugs (rifampicin and isoniazid) and to some second-line molecules in its latest version, are often used as substitutes for conventional drug susceptibility testing, although their implementation is still challenged by high costs and complex technical requirements.^41,42^ The MTBDRsl molecular test has been recently endorsed by WHO.^43^ Whole-genome sequencing is under investigation as a potentially promising tool to detect relevant mutations that could predict drug resistance and help to design individualised therapeutic regimens.^44^

Tuberculin skin test and interferon-gamma release assay are currently recommended for the diagnosis of LTBI, but have a marginal role in the detection of TB cases, as they cannot distinguish between active and latent infection and may be influenced by the patient’s immune status. These tests will be further discussed in another section of this paper.

A relevant proportion of the estimated 9.6 million TB cases worldwide is not detected either because of limited access to healthcare facilities or because of the unavailability of appropriate diagnostic methods. Reaching the ‘missed’ 3 million cases is among the top five priorities to eliminate TB, and to do so, new point-of-care tests are urgently needed.^45^ Research is ongoing to develop a diagnostic tool that meets the basic characteristics of rapidity, low cost, high sensitivity and technical simplicity. There is increasing interest in the mycobacterial lipoarabinomannan (LAM) urinary detection method (and its ‘lateral flow’ variant) that may be very useful in diagnosing active TB. LAM is an antigen located in mycobacterial outer cell wall, and it is thought to be present in individuals with active disease but not in those with LTBI, irrespective of their immunological conditions.^46–48^ This test, that can be easily performed in peripheral centres leading to better access to care, may prove advantageous for ruling out TB in children (given the simplicity of urine collection compared with other clinical samples) and facilitate the identification of extrapulmonary forms of TB. Unfortunately, no information is provided about the mycobacterial species and the drug susceptibility pattern of the infecting strain that is a considerable limit when dealing with high-risk populations and/or in high MDR-TB-burden contexts. Based on the current evidence, the WHO does not recommend the use of urinary LAM detection in patients with presumptive TB with the exception of HIV-positive individuals with low CD4+ counts or who are seriously ill, who may benefit from this test in addition to a well-established diagnostic tool.^49^ Other studies are underway to investigate on the role of mycobacterial metabolic products in exhaled breaths (the so-called ‘volatile organic compounds’) in the diagnosis of TB in symptomatic patients.^50^

It is of paramount importance that general practitioners are familiar with the standards for diagnosis of TB, as the ‘dangerous’ case of TB is the undetected case, and rapid diagnosis (coupled with effective treatment) is the essence of TB control.^30,31^

## TB treatment

Anti-TB treatment aims to cure the patient, prevent complications and death, avoid relapses, reduce the transmission potential to susceptible individuals, and limit the emergence and spread of drug-resistant strains. For all these reasons, the therapeutic approach to TB requires the use of multiple drugs. Treatment should include an intensive phase aimed at markedly decreasing the bacterial burden, followed by a ‘sterilising’ consolidation phase, with an overall duration of at least 6 months. Longer treatments may be required in selected situations, such as in patients with extensive bone involvement or those with cerebral tuberculomas.^51^ The first-line standard regimen that is currently recommended for drug-susceptible TB is based on a 2-month intensive phase with four drugs (isoniazid, rifampicin, pyrazinamide and ethambutol; HRZE) followed by a 4-month consolidation phase with two drugs (isoniazid and rifampicin; HR). Dose adjustment is required for children according to body weight, but the regimen composition remains the same.^52^ Comorbidities do not justify any changes in the therapeutic approach to TB, although potential drug–drug interactions should be carefully evaluated and managed if necessary. Optimal adherence throughout the whole duration of treatment is crucial, as poor compliance is among the major causes of treatment failure, being associated with a high risk of resistance selection. Hospitalisation of drug-susceptible TB patients is usually unnecessary, except for those presenting with severe clinical manifestations or in case of significant social fragility, which may considerably interfere with monitoring and compliance.

Although the treatment success rate of drug-susceptible TB exceeds 85% at global level even in high-burden settings, the outcome is much poorer for patients with drug-resistant TB (50% on average).^1^ MDR-TB is defined as resistance to at least rifampicin and isoniazid, whereas XDR-TB includes MDR-TB cases with additional resistances to any fluoroquinolone and any of the injectable drugs. The treatment outcomes are lower in XDR-TB cases (40% on average), but they may be as low as 19% when the disease is sustained by MTB strains with a resistance pattern beyond XDR.^53,54^

A revision of the former classification of anti-TB drugs in five different groups (i.e., all first-line agents belonged to group 1, whereas groups 2–5 contained second-line molecules characterised by lower efficacy and a less favourable safety profile) has been recently approved based on the current evidence on each drug (Table 1).^43,55,56^ The conventional regimen for rifampicin resistant or MDR-TB currently recommended by the WHO consists of at least five effective anti-TB drugs during the intensive phase, including pyrazinamide and four core second-line anti-TB drugs (one chosen from group A, one from group B and at least two from group C2; if the minimum number of effective anti-TB drugs cannot be reached by using agents from groups A to C alone, drugs from group D2 or, if not possible, from group D3 are added to bring the total to five); agents from group D1 are added if they are considered to add benefit (e.g., high-dose isoniazid in patients without high-level isoniazid resistance; Table 2).

Up to now, a total treatment duration of 18–20 months has been recommended for most MDR-TB patients, but the optimal duration of treatment is actually a matter of debate.^55^ Shorter regimens (lasting <12 months) based on a combination of likely effective second-line drugs have recently been evaluated, first in Bangladesh and then in other countries (mostly in Africa), showing very promising results.^57–59^ The WHO guidelines have been updated in May 2016 in accordance with these findings, including a recommendation for the use of shorter treatment options if specific conditions are met.^43^ In particular, patients who were previously exposed to second-line agents, pregnant women, extrapulmonary TB cases, XDR-TB patients or those with intolerance to any of the regimen components cannot be considered eligible. Given that treatment of M/XDR-TB is very expensive, a significant reduction of costs and a higher rate of patients’ retention on care are among the major advantages of this innovative approach.^60,61^ However, the correct management of such regimens is pivotal in order not to promote the selection of further resistances, which makes it even more important to reserve M/XDR-TB case management only to highly experienced clinicians based on international guidelines.^62,63^

Over a 40-year period, only two new molecules (bedaquiline and delamanid) have been developed and approved by WHO for selected cases.^64–68^ Some other drugs (such as carbapenems and linezolid) have been recently repurposed for the use in M/XDR-TB treatment and are currently classified as group 5 drugs.^69–73^ All these agents require a careful management in the context of individualised regimens under close clinical and laboratory monitoring.^62^

WHO and partners have devoted specific attention to the management of paediatric TB in the last few years because of the specific difficulties in diagnosing and managing treatment in this specific population.^52^

Although no recommendations have been issued up to now for the use of bedaquiline and delamanid in the paediatric population due to the lack of scientific evidence (controlled clinical trials are still ongoing), encouraging findings on delamanid have emerged from some pharmacokinetic studies conducted on children over 6 years of age.^74–76^

To our knowledge, co-administration of these two new drugs in the same patient has only been described for sporadic adult cases, and WHO is not recommending their association as evidence does not allow it yet.^77–79^ Rigorous criteria have been recommended to ensure the appropriate management of such regimens in well-recognised reference centres.^63^

Therapeutic drug monitoring has been proposed as a potentially useful tool for treatment individualisation especially for selected patient categories such as those at high risk for drug–drug interactions or drug malabsorption and/or under-dosing, thus limiting the risk of adverse events and leading to a reduction of costs.^60,61^ However, a standardised protocol has not been defined yet and further investigations are needed to better assess the role of therapeutic drug monitoring in the routine clinical practice.^80–86^ Nevertheless, designing the best therapeutic regimen for a single patient is likely to remain quite a challenging task until more effective and less toxic drugs are not be available.^87^

In the complex scenario of the M/XDR-TB epidemic, a web-based free-of-charge tool jointly developed by the European Respiratory Society and the WHO was launched in 2012 to help clinicians facing the challenges of drug resistance management by providing tailored expert opinion on difficult-to-treat cases (www.tbconsilium.org).^88^

As any TB patient is potentially infectious till appropriate treatment has been initiated, appropriate infection control measures need to be ensured, through managerial, administrative and environmental interventions.^89–91^ A recent European study has shown that even in qualified centres, infection control practices are sub-optimally applied, further contributing to circulation of MTB among other patients, staff, visitors and ultimately within the community.^89–91^

The contribution of general practitioners in ensuring correct treatment of TB cases, in collaboration with the specialists who usually initiate it, is of paramount importance, due to the special relationship they have with their patients and the possibility to educate, foster adherence, monitoring treatment and ensuring directly observed therapy.^30,31^

## LTBI and TB prevention

The natural history of TB begins with the acquisition of infection through the inhalation of MTB that subsequently enters a replication cycle and disseminates within the host, whose immune system usually reacts and leads to containment of viable bacilli. The result of this process is asymptomatic LTBI, a condition in which TB bacteria remain in the body in a quiescent state, without making any damage and therefore without causing transmission to other susceptible individuals.^92^

Although it is substantially impossible to test everybody, mathematical models estimate that about a third of the global population has LTBI, an incredibly large pool from which TB cases actually arise each year.^93^

Most people remain latently infected for their whole life, often unaware of their condition, and do not constitute a public health problem. However, ~5–15% of them may experience reactivation at some point in time, thus becoming TB cases with transmission potential. It is thought that reactivation actually leads to as many as 80% of the global TB cases, which is particularly true in low-incidence countries. The risk of progression from latent infection to active disease is considerably higher in some populations that therefore requires special attention and targeted interventions.^94^ The management of LTBI is now fully recognised as a core component of the End-TB Strategy, as demonstrated by the recent development of specific guidelines by WHO.^95^ Among the main objectives of this document is the definition of the so-called ‘high-risk groups’, i.e., those who are more likely to undergo TB reactivation. People living with HIV and children under 5 years of age who are household or close contacts of a TB case represent the priority target, being the most vulnerable categories in any setting. All such individuals should be systematically put on LTBI treatment whenever active TB is excluded.^52,96–98^ Other populations should be carefully considered according to the local resources in high- and upper middle-income countries with low TB incidence; they include candidates to tumour necrosis factor-alpha inhibitor treatment, patients with silicosis, transplant recipients and patients requiring haemodialysis, for whom systematic testing and treatment is strongly recommended.^95^ In addition to those mentioned above, other at-risk groups may be identified such as migrants from high TB-burden countries, prisoners, healthcare workers, homeless people or injection drug users, who may also benefit from LTBI treatment if feasible. Current diagnostic tests do not allow direct identification of dormant TB microorganisms in a latently infected subject. For this reason, LTBI can only be diagnosed by evaluating the immunological response to *in vivo* or *in vitro* stimulation by mycobacterial antigens, which is the underlying principle of the tuberculin skin test and interferon-gamma release assays.

According to current recommendations, tuberculin skin test and interferon-gamma release assays can be considered interchangeable, and tuberculin skin test should not replace interferon-gamma release assays for LTBI diagnosis in low- and middle-income settings. Both tests are limited by the inability to predict the risk of reactivation and cannot distinguish between latent infection and active disease.^95,99^ In case of a positive result to any of them, active TB must be reasonably ruled out before starting LTBI treatment, which is essential to avoid the selection of drug resistances. A 6-month course of daily isoniazid is the only currently recommended option for treatment in resource-limited settings, with life-long administration encouraged among people living with HIV in high TB transmission areas.^96,100^ Alternative therapeutic regimens can be considered in high-income countries for patients other than children and HIV-positive individuals (Tables 3 and 4).^95^

## Concluding remarks

Current data on TB epidemiology suggest that targeted sensitisation initiatives are still largely needed to raise awareness among clinicians on the importance of this disease. Thinking about TB is the first step to fight TB, as early diagnosis and prompt initiation of treatment are essential to achieve the best outcome and limit the risk of transmission to other individuals. Special attention should be paid to the so-called ‘high-risk populations’, who are more likely to be infected with TB bacteria and to develop an active disease, thus maintaining the vicious cycle of transmission within the community. Primary care providers are the key to both control and elimination strategies, which, at the end, are based on correct diagnosis and treatment of TB patients and latently infected individuals.

Primary healthcare providers, in fact, have a crucial role in the identification of at-risk subjects who may benefit from LTBI screening and treatment, as well as of TB suspects who should be referred to specialised centres for further investigations and management. Empirical use of anti-TB drugs outside the context of reference TB care facilities should be strongly discouraged to avoid the selection of drug resistances, which makes the therapeutic management of affected patients much more challenging.

## Figures and Tables

**Table 1:** Anti-TB drugs recommended for the treatment of rifampicin-resistant and multidrug-resistant TB^a^ (from ref. 43)

*Group name*	*Anti-tuberculosis drugs*	*Abbreviation*
A. Fluoroquinolones^b^	Levofloxacin	Lfx
	Moxifloxacin	Mfx
	Gatifloxacin	Gfx
		
B. Second-line injectable agents	Amikacin	Am
	Capreomycin	Cm
	Kanamycin	Km
	(Streptomycin)^c^	(S)
		
C. Other core second-line agents^b^	Ethionamide/Prothionamide	Eto/Pto
	Cycloserine/Terizidone	Cs/Trd
	Linezolid	Lzd
	Clofazimine	Cfz
		
*D. Add-on agents (not part of the core MDR-TB regimen)*		
D1	Pyrazinamide	Z
	Ethambutol	E
	High-dose isoniazid	Hh
D2	Bedaquiline	Bdq
	Delamanid	Dlm
D3	*p*-Aminosalicylic acid	PAS
	Imipenem cilastatin^d^	Ipm
	Meropenem^d^	Mpm
	Amoxicillin clavulanate^d^	Amx-Clv
	(Thioacetazone)^e^	(T)

a This regrouping is intended to guide the design of conventional regimens; for shorter regimens lasting 9–12 months, the composition is usually standardised.

b Medicines in groups A and C are shown by decreasing order of usual preference for use.

c Although streptomycin is not usually included with the second-line drugs, it can be used as the injectable agent of the core MDR-TB regimen if none of the three other agents can be used and if the strain can be reliably shown not to be resistant. Resistance to streptomycin alone does not qualify for the definition of extensively drug-resistant TB (XDR-TB).

d Carbapenems and clavulanate are meant to be used together; clavulanate is only available in formulations combined with amoxicillin.

e HIV status must be tested and confirmed to be negative before thioacetazone is started.

**Table 2:** Conventional treatment regimens for rifampicin-resistant or MDR-TB (adapted from ref. 43)

*World Health Organization recommendations*
In patients with RR or MDR-TB, a regimen with at least five effective anti-TB during the intensive phase is recommended, including:
• Pyrazinamide
• Four core second-line anti-TB drugs:
One from group A (Lfx, Mfx, Gfx)
One from group B^a^ (Am, Cm, Km, (S))
At least two from group C (Eto/Pto, Cs/Trd, Lzd, Cfz)

If the minimum of effective TB medicines cannot be composed as above, an agent from group D2 (Bdq, Dlm) and other agents from D3 (PAS, Ipm, Mpm, Amx-Clv, T) may be added to bring the total to five.

In patients with RR or MDR- TB, it is recommended that the regimen be further strengthened with high-dose isoniazid and/or ethambutol

It is recommended that any patient—child or adult—with RR-TB in whom isoniazid resistance is absent or unknown be treated with a recommended MDR-TB regimen, either a shorter MDR-TB regimen, or if this cannot be used, a conventional MDR-TB regimen to which isoniazid is added.

Abbreviations: MDR-TB, multidrug-resistant tuberculosis; RR, rifampicin resistant.

a In children with non-severe disease, group B anti-TB drugs may be excluded.

**Table 3:** Recommended treatment options for LTBI (adapted from ref. 95)

*Treatment options for LTBI*
6-month isoniazid
or 9-month isoniazid
or 3-month regimen of weekly rifapentine plus isoniazid^a^
or 3–4 months isoniazid plus rifampicin^a^
or 3–4 months rifampicin alone^a^

a Rifampicin- and rifapentine-containing regimens should be prescribed with caution to people living with HIV who are on antiretroviral treatment due to potential drug-to-drug interactions.

**Table 4:** Recommendations for LTBI screening and treatment in different high-risk population groups in high- and upper middle-income countries with low TB incidence (adapted from ref. 95)

*In high-income and upper middle-income countries with estimated TB incidence* <*100 per 100,000 population*
• Systematic testing and treatment of LTBI should be performed in people living with HIV, adult and child contacts of pulmonary TB cases, patients initiating anti-tumour necrosis factor (TNF) treatment, patients receiving dialysis, patients preparing for organ or haematologic transplantation, and patients with silicosis. Either interferon-gamma release assays (IGRAs) or Mantoux tuberculin skin test (TST) should be used to test for LTBI.

• Systematic testing and treatment of LTBI should be considered for prisoners, health workers, immigrants from high TB-burden countries, homeless persons and illicit drug users. Either IGRA or TST should be used to test for LTBI.

• Systematic testing for LTBI is not recommended in people with diabetes, people with harmful alcohol use, tobacco smokers and in underweight people unless they are already included in the above recommendations.

*For resource-limited countries and other middle-income countries that do not belong to the above*
• People living with HIV and children below 5 years of age who are household or close contacts of people with TB and who, after an appropriate clinical evaluation, are found not to have active TB but have LTBI should be treated.
